# SARS-CoV-2 receptor binding domain displayed on HBsAg virus–like particles elicits protective immunity in macaques

**DOI:** 10.1126/sciadv.abl6015

**Published:** 2022-03-16

**Authors:** Neil C. Dalvie, Lisa H. Tostanoski, Sergio A. Rodriguez-Aponte, Kawaljit Kaur, Sakshi Bajoria, Ozan S. Kumru, Amanda J. Martinot, Abishek Chandrashekar, Katherine McMahan, Noe B. Mercado, Jingyou Yu, Aiquan Chang, Victoria M. Giffin, Felix Nampanya, Shivani Patel, Lesley Bowman, Christopher A. Naranjo, Dongsoo Yun, Zach Flinchbaugh, Laurent Pessaint, Renita Brown, Jason Velasco, Elyse Teow, Anthony Cook, Hanne Andersen, Mark G. Lewis, Danielle L. Camp, Judith Maxwell Silverman, Gaurav S. Nagar, Harish D. Rao, Rakesh R. Lothe, Rahul Chandrasekharan, Meghraj P. Rajurkar, Umesh S. Shaligram, Harry Kleanthous, Sangeeta B. Joshi, David B. Volkin, Sumi Biswas, J. Christopher Love, Dan H. Barouch

**Affiliations:** 1 Department of Chemical Engineering, Massachusetts Institute of Technology, Cambridge, MA 02139, USA.; 2 The Koch Institute for Integrative Cancer Research, Massachusetts Institute of Technology, Cambridge, MA 02139, USA.; 3 Center for Virology and Vaccine Research, Beth Israel Deaconess Medical Center, Harvard Medical School, Boston, MA 02215, USA.; 4 Department of Biological Engineering, Massachusetts Institute of Technology, Cambridge, MA 02139, USA.; 5 Department of Pharmaceutical Chemistry, Vaccine Analytics and Formulation Center, University of Kansas, Lawrence, KS 66047, USA.; 6 Departments of Infectious Diseases and Global Health and Comparative Pathobiology, Cummings School of Veterinary Medicine, Tufts University, North Grafton, MA 01536, USA.; 7 Harvard Medical School, Boston, MA 02115, USA.; 8 SpyBiotech Limited, Oxford Business Park North, Oxford OX4 2JZ, UK.; 9 Bioqual, Rockville, MD 20852, USA.; 10 Bill and Melinda Gates Medical Research Institute, Cambridge, MA 02139, USA.; 11 Serum Institute of India Pvt. Ltd., Pune, India.; 12 Bill and Melinda Gates Foundation, Seattle, WA 98109, USA.; 13 The Jenner Institute, University of Oxford, Oxford OX3 7DQ, UK.; 14 Ragon Institute of MGH, MIT, and Harvard, Cambridge, MA 02139, USA.; 15 Massachusetts Consortium on Pathogen Readiness, Boston, MA 02115, USA.

## Abstract

Authorized vaccines against SARS-CoV-2 remain less available in low- and middle-income countries due to insufficient supply, high costs, and storage requirements. Global immunity could still benefit from new vaccines using widely available, safe adjuvants, such as alum and protein subunits, suited to low-cost production in existing manufacturing facilities. Here, a clinical-stage vaccine candidate comprising a SARS-CoV-2 receptor binding domain–hepatitis B surface antigen virus–like particle elicited protective immunity in cynomolgus macaques. Titers of neutralizing antibodies (>10^4^) induced by this candidate were above the range of protection for other licensed vaccines in nonhuman primates. Including CpG 1018 did not significantly improve the immunological responses. Vaccinated animals challenged with SARS-CoV-2 showed reduced median viral loads in bronchoalveolar lavage (~3.4 log_10_) and nasal mucosa (~2.9 log_10_) versus sham controls. These data support the potential benefit of this design for a low-cost modular vaccine platform for SARS-CoV-2 and other variants of concern or betacoronaviruses.

## Teaser

A modular protein subunit vaccine candidate produced in yeast confers protection against SARS-CoV-2 in nonhuman primates.

## INTRODUCTION

Prophylactic vaccination is effective in eliciting protective immunity against severe acute respiratory syndrome coronavirus-2 (SARS-CoV-2) and preventing coronavirus disease 2019 (COVID-19) (*1*). Multiple vaccines have now been distributed at large scale in many countries and have resulted in a lower incidence of infection and severe disease caused by SARS-CoV-2 (*2*, *3*). Access to vaccines remains limited, however, in low- and middle-income countries (LMICs), where infectious variants of SARS-CoV-2 continue to emerge in large-scale outbreaks (*4*). In addition to financial and logistical support from developed countries and health organizations, vaccines produced by local manufacturers could enable the lowest costs for interventions in these countries and potentially minimize the infrastructure required for their distribution (*5*–*7*). Protein subunit vaccines are a promising solution because they can be manufactured using existing large-scale microbial fermentation facilities in LMICs (*8*), typically do not require frozen storage and distribution, and are safe and effective when used with adjuvants (*9*, *10*).

We sought to design a protein subunit vaccine that would be both suitably immunogenic and simple to manufacture for affordable distribution in LMICs. Multiple protein vaccines based on the trimeric SARS-CoV-2 spike protein have demonstrated efficacy but are manufactured in insect or mammalian cells, which are difficult to transfer to existing facilities in LMICs (*11*, *12*). The receptor binding domain (RBD) of the spike protein has been proposed as an alternative to the full spike protein because it has been shown to elicit multiple potent neutralizing antibodies directed at multiple epitopes (*13*–*15*) and can be manufactured in microbial systems like the biotechnological yeast *Komagataella phaffii* (*Pichia pastoris*) (*16*, *17*). Formulations comprising only monomeric RBD (Wuhan-Hu-1) and adjuvant tested to date elicit lower titers of neutralizing antibodies in humans after three doses when compared to vaccines based on the full spike protein (*18*–*20*). While further optimization of such formulations could improve these designs, we and others have demonstrated that multimeric display of RBD on virus-like particles (VLPs) can be highly immunogenic and comparable to vaccines based on the full spike protein (*21*–*26*).

Here, we describe the design and immunogenicity of a modular protein subunit vaccine, comprising a SARS-CoV-2 spike protein subunit RBD displayed on a hepatitis B VLP that is constructed using a covalent peptide–mediated linkage (SpyTag/SpyCatcher). Both of these vaccine components are currently produced by microbial fermentation at a large-scale manufacturing facility in India. We show that this vaccine candidate elicits a strong immune response in cynomolgus macaques and protects against SARS-CoV-2 challenge. On the basis of these promising data, this vaccine candidate is currently being tested in clinical trials (Australian New Zealand Clinical Trials Registry registration number ACTRN12620000817943).

## RESULTS

### Design of an accessible protein subunit vaccine

We selected hepatitis B surface antigen (HBsAg) VLPs as a nanoparticle on which to display the RBD. This choice leverages extensive experience with a previously tested commercial product, GeneVac-B, that is manufactured at low cost and distributed in LMICs for prevention of hepatitis B (*27*). As a model for our design here, we referenced previously reported designs with this core nanoparticle decorated with a malarial subunit antigen (*28*). A polypeptide-based system (SpyTag/SpyCatcher) allowed for covalent linkage of the antigen to the VLP by a transpeptidation reaction. This general design can increase antigen-specific antibody titers in mice, and the responses elicited in prior examples were unaffected by the presence of preexisting antibodies against HBsAg (*29*). The modularity of the SpyTag/SpyCatcher system allows each component of the final particle to be expressed and purified independently to maximize yields and quality.

**Fig. 1.**
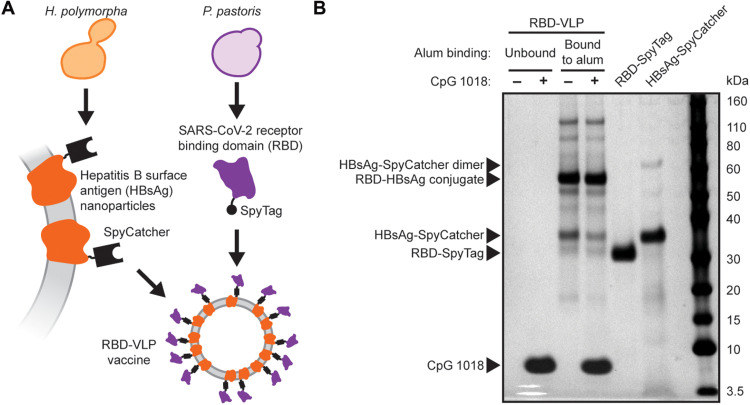
Design and analysis of the RBD-VLP drug product. (**A**) Schematic of protein expression and conjugation. (**B**) Reduced SDS-PAGE analysis of the formulated RBD-VLP vaccine samples. Alum-bound protein antigen (with and without CpG) was separated by centrifugation and desorbed from the alum using an elution buffer combined with heat treatment before SDS-PAGE.

We adapted this approach to make a vaccine candidate for COVID-19. We genetically fused the SpyTag peptide onto the SARS-CoV-2 RBD. This fusion protein was manufactured in an engineered strain of *K. phaffii* (*30*). The RBD-SpyTag and HBsAg-SpyCatcher VLPs were each purified separately and then conjugated in a Good Manufacturing Process (GMP) process to produce the RBD-VLP antigen (Fig. 1A). In this study, the RBD-VLP antigen was formulated with two adjuvants: (i) aluminum hydroxide (alum) and (ii) alum combined with CpG 1018—a potent Toll-like receptor 9–agonizing adjuvant known to elicit T helper cell 1 (T_H_1)–like responses (*31*) and used in the commercial hepatitis B vaccine, HEPLISAV-B. Analysis of the formulated vaccine drug product by SDS–polyacrylamide gel electrophoresis (SDS-PAGE) showed only small fractions (<20%) of unconjugated HBsAg-VLP and RBD and complete adsorption of the RBD-VLP antigen onto the alum adjuvant (Fig. 1B). We detected CpG 1018 in both the unbound and bound to alum fractions.

We also performed additional analytics on unformulated RBD-VLP antigen to confirm antigenicity and nanoparticle formation. The RBD-VLP antigen exhibited strong binding to the human Angiotensin-converting enzyme 2 (ACE2) receptor and a known neutralizing antibody CR3022 by biolayer interferometry (fig. S1A). The large difference in signal observed in this analysis between the RBD-VLP and soluble monomeric RBD-SpyTag confirms the multivalency of the RBD conjugated on the VLP. We also confirmed formation of nanoparticles by electron microscopy (EM) (fig. S1, B and C). These analytics confirmed the structural attributes of the conjugated RBD-VLPs used for nonclinical evaluations here.

### Immunogenicity testing in cynomolgus macaques

**Fig. 2.**
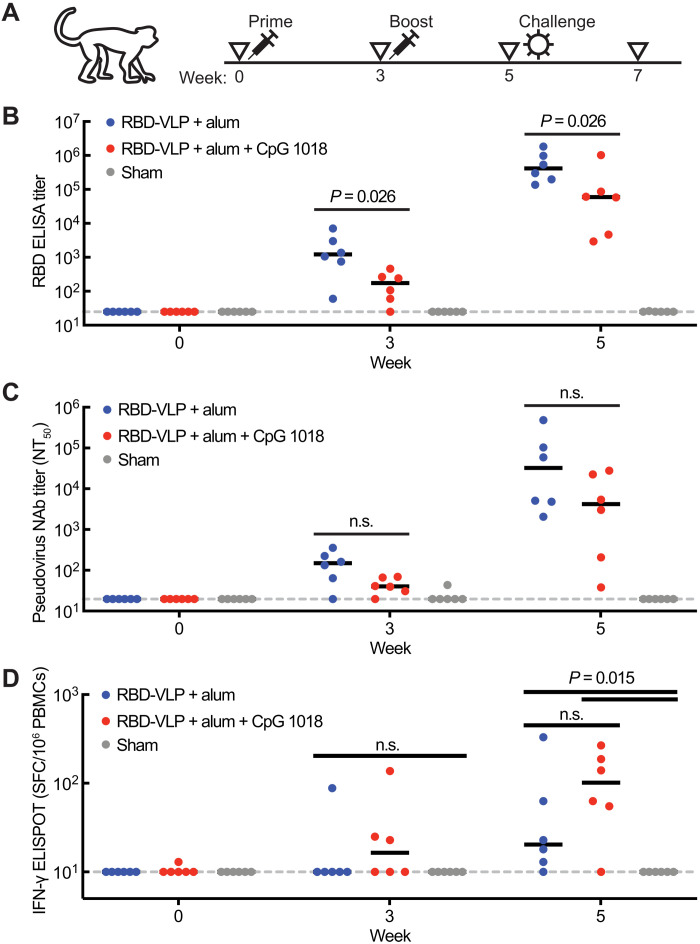
Humoral and cellular immune response to the RBD-VLP vaccine. (**A**) Design of the nonhuman primate study in cynomolgus macaques. (**B**) Titers of RBD-specific antibody binding in animal sera. (**C**) Titers of SARS-CoV-2 pseudovirus–neutralizing antibody in animal sera. NT_50_, end point of 50% reduction of virus expression. (**D**) Expression of IFN-γ from cells stimulated with S1 protein peptides. Statistical significance was determined by a Kolmogorov-Smirnov test. n.s., not significant (*P* > 0.1). Black bars represent median values. Gray dotted line represents limit of detection. PBMCs, peripheral blood mononuclear cells.

To assess the immunogenicity of this RBD-VLP vaccine candidate, we immunized three groups of six cynomolgus macaques with two doses of either vaccine formulation (alum or alum combined with CpG 1018) or a placebo, spaced 3 weeks apart (Fig. 2A). We assessed spike protein–specific antibody titers after each dose (Fig. 2B). We observed full seroconversion and high antibody titers for both RBD-VLP vaccine formulations. Formulation with only alum elicited significantly higher-binding antibody titers. We assessed the neutralizing activity of the sera against a SARS-CoV-2 pseudovirus and observed high titers of neutralizing antibodies for both formulation with alum only and formulation with alum and CpG 1018 (Fig. 2C). Formulation with only alum appeared to yield higher neutralization but was not significantly higher (*P* > 0.1, Kolmogorov-Smirnov test) than the vaccine formulated with both alum and CpG 1018. Neutralization of SARS-CoV-2 pseudovirus correlated well with spike protein–specific antibody titer (fig. S2A).

We also assessed the neutralizing activity of sera from week 5 against SARS-CoV-2 pseudoviruses with spike proteins from several circulating variants of concern (fig. S2B). Sera from animals immunized with RBD-VLP in both vaccine formulations exhibited similar neutralizing activities against D614G and B.1.1.7 variants. While changes in the neutralizing activities of sera from animals immunized with RBD-VLP with alum and CpG 1018 against B.1.351 were not statistically significant, the neutralizing activities of sera from animals immunized with RBD-VLP with only alum were significantly reduced against B.1.351 (~25×, geometric mean).

To determine why the formulation with only alum elicited higher antibody titers, we assessed the antigenicity for retained samples of the RBD-VLP in both vaccine formulations used. We observed ~30% less binding to human ACE2 for the alum and CpG 1018 formulation compared to the formulation with alum only [competitive enzyme-linked immunosorbent assay (ELISA)] (fig. S2C). The formulation with alum and CpG 1018 at the concentrations used here appears to either have altered the antigenicity of the RBD-VLP antigen or destabilized the SpyTag/SpyCatcher-mediated linkage of RBD and VLP, potentially leading to a reduced humoral immune response.

After assessing the humoral response to the RBD-VLP vaccine candidate, we profiled the cellular immune response. We assessed expression of interferon-γ (IFN-γ) in cells stimulated with peptides from the S1 region of the spike protein, which includes the RBD (Fig. 2D). We observed significant IFN-γ expression in cells from both the alum formulation and the alum and CpG 1018 formulation after two doses. The cellular response appeared stronger with the alum and CpG 1018 coformulation, consistent with previous reports on the influence of CpG 1018 as an adjuvant in vaccines (*31*), but the effect was not significant (*P* > 0.4, Kolmogorov-Smirnov test).

### Challenge with SARS-CoV-2

**Fig. 3.**
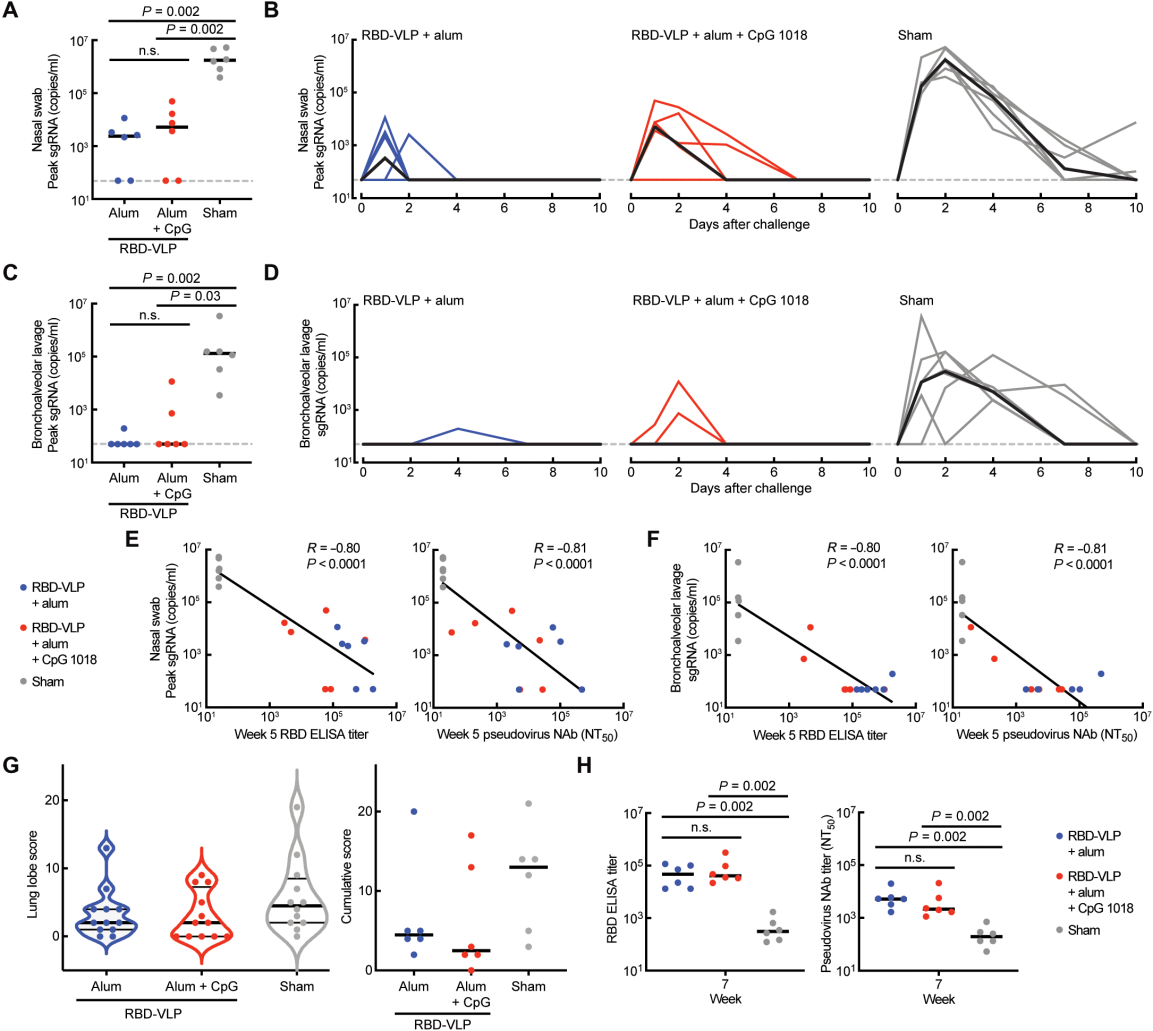
Challenge with SARS-CoV-2. (**A** and **C**) Peak levels of SARS-CoV-2 sgRNA after challenge for each animal from nasal swabs (A) or BAL (C). Statistical significance was determined by a Kolmogorov-Smirnov test. n.s., not significant (*P* > 0.1). Black bars represent median values. Gray dotted line represents limit of detection. (**B** and **D**) Levels of sgRNA after challenge from nasal swabs (B) or BAL (D). Each thin line represents one animal. Thick black lines represent median values. (**E** and **F**) Correlation of RBD-specific antibody titer and pseudovirus-neutralizing antibody titer from week 5 animal sera with peak sgRNA levels in nasal swabs (E) and BAL (F). *R* was calculated by Spearman correlation. (**G**) Pathology scores for individual lung samples and cumulative scores for each animal. (**H**) Titers of RBD-specific antibody and SARS-CoV-2 pseudovirus–neutralizing antibody in animal sera at week 7 (2 weeks after challenge). Statistical significance was determined by a Kolmogorov-Smirnov test. n.s., not significant (*P* > 0.1). Black bars represent median values.

To assess whether the RBD-VLP vaccine could protect animals from infection, we challenged all animals with SARS-CoV-2 2 weeks after the second immunization (Fig. 2A). We monitored the course of infection for 2 weeks by measuring titers of subgenomic RNA (sgRNA) in nasal swabs and bronchoalveolar lavage (BAL) supernatants. Both formulations of the RBD-VLP vaccine significantly reduced the levels of detected sgRNA in the upper respiratory tract (Fig. 3A), and no sgRNA was detected on day 4 after challenge from the group immunized with RBD-VLP formulated with alum (Fig. 3B). Both RBD-VLP formulations exhibited nearly complete protection from viral infection in the lower respiratory tract—sgRNA was detected in BAL supernatants from only three animals (Fig. 3, C and D). We observed significant correlation between prechallenge antibody titers and measured sgRNA levels (Fig. 3, E and F).

We also assessed lung samples from each animal after challenge with SARS-CoV-2 and scored each sample for histopathological findings. Animals that received the sham vaccine showed evidence of interstitial inflammation, syncytial cells, and type II pneumocyte hyperplasia in the lung, consistent with SARS-CoV-2 replication and a higher median cumulative pathology score (*13*) as compared to immunized animals (median, 2.5 to 4.5) (Fig. 3G).

Last, we measured RBD-specific and pseudovirus-neutralizing antibody titers after challenge. Animals that received the RBD-VLP vaccine candidate had higher antibody levels than animals that were not immunized prechallenge (Fig. 3H). Notably, postchallenge levels of humoral immunity conferred by the vaccine were not significantly different between the two vaccine formulations (*P* > 0.4, Kolmogorov-Smirnov test). These results together suggest that the RBD-VLP vaccine candidate can protect from SARS-CoV-2 infection.

## DISCUSSION

In this study, we report the design of an RBD-VLP–based protein subunit vaccine candidate for COVID-19, currently manufactured in existing production facilities at the Serum Institute of India. Both protein components are produced in yeast, making this vaccine a promising, low-cost intervention for LMICs.

We tested the vaccine in cynomolgus macaques, which have been shown to be equivalent to rhesus macaques as a model for SARS-CoV-2 infection (*32*). The RBD-VLP vaccine elicited high titers of neutralizing antibodies (>10^4^) that were above the range of protection for other licensed vaccines in nonhuman primates (*33*, *34*). The vaccine also conferred protection from viral loads in the upper and lower respiratory tract upon challenge with SARS-CoV-2.

CpG adjuvants with HBsAg vaccines have been shown to boost the T_H_1 cell response to complement the T_H_2 cell response induced with alum alone (*31*, *35*, *36*). In agreement with these prior reports, we did observe a higher T_H_1 cell response to the RBD-VLP formulated with both alum and CpG 1018 compared to alum only. We also observed, however, a reduced humoral immune response when the vaccine was formulated with alum and CpG 1018, while the results were not significant. We attributed this difference to a reduction in antigenicity of the RBD-VLP; inclusion of CpG 1018 may destabilize the SpyTag/SpyCatcher binding or affect the structural integrity of the RBD, leading to altered antigenicity. Another multimeric RBD vaccine has reported robust humoral responses in nonhuman primates with the same dose (1500 μg) of CpG 1018 (*25*), but in that instance, the vaccine was formulated 30 min before injection. This discrepancy suggests that a high dose of CpG 1018 may affect the antigenicity of the RBD or destabilize the SpyTag/SpyCatcher binding when stored for weeks or months at 4°C before use. Reduced dosing of CpG 1018 could lessen the impact on the stability of the RBD-VLPs while still eliciting balanced T_H_1 and T_H_2 cellular responses (*37*). This question warrants further study to define the underlying mechanism and optimize the concentrations of this coadjuvant in formulations of vaccine candidates to balance the stability of the RBD-VLP antigen with the beneficial effects of the alum and CpG 1018 adjuvants. Clinical trials of this RBD-VLP vaccine candidate to date have been formulated with only alum.

Another potential challenge posited for CoVID-19 and interventions directed toward SARS-CoV-2 is the possibility of antibody-dependent enhancement or vaccine-associated enhanced disease (*38*). Such effects could present safety concerns for novel vaccines (*39*). Both vaccine candidates tested here with alum or CpG 1018 and alum as adjuvants did not show evidence for disease enhancement following vaccination and subsequent challenge. This outcome is consistent with evidence that passive immunization with human antibodies directed against SARS-CoV-2 have not exhibited disease enhancement in mice and monkeys (*40*) and provides support that formulations of the vaccine here with alum alone could provide protective immunity without disease enhancement.

The report of this vaccine comes at a befitting time in the COVID-19 pandemic. Variants of SARS-CoV-2 with increased transmissibility continue to drive outbreaks of COVID-19 in LMICs. Vaccines based on mRNA have been shown to be effective against emerging variants (*41*, *42*) but remain largely inaccessible in Southeast Asia and Africa due to high costs and cold-chain requirements. Responses induced by currently approved protein-based vaccines based on the Wuhan-Hu-1 strain, on the other hand, have so far been less effective in neutralizing activity exhibited with sera against circulating variants (*43*). Encouragingly, vaccines based on the RBD alone effectively boost an immune response originally generated against a full-length spike protein trimer (*44*). There is growing interest, therefore, in the utility of RBD-based vaccine boosters to provide immunity against emerging variants of SARS-CoV-2 that may escape antibody responses induced by vaccination against the Wuhan-Hu-1 or D614G spike protein.

The RBD-VLP vaccine candidate presented here is modular and could be manufactured and formulated with RBD antigens from emerging variants or with engineered immunogenicity (*26*). We reported previously that the RBDs from the B.1.1.7 and B.1.351 variants can be manufactured from the same microbial strains and fermentation processes (*30*). A mosaic vaccine against multiple strains of SARS-CoV-2 could be simply produced by conjugation of multiple RBD antigens to the HBsAg-VLP. This approach could also be tailored to generate multivalent designs for a panel of SARS-CoV-2 variants or for broadly targeting betacoronaviruses (*23*). This modular design of protein antigens paired with existing large-scale manufacturing capacity in yeast demonstrates a vaccine platform that could keep pace with an evolving COVID-19 pandemic and provide better access to COVID-19 vaccines around the globe.

## MATERIALS AND METHODS

### Production of vaccine samples

HBsAg SpyCatcher nanoparticles and RBD-SpyTag protein were produced by yeast fermentation in a GMP process at the Serum Institute of India Pvt. Ltd. Samples were stored at 4°C for several weeks before immunization.

### SDS–polyacrylamide gel electrophoresis

Formulated RBD-VLP samples were centrifuged at 4000*g* for 10 min at 4°C. The supernatant with unbound protein was transferred to another tube without disturbing the pellet and prepared for SDS–polyacrylamide gel electrophoresis (SDS-PAGE) in 50 mM dithiothreitol (Thermo Fisher Scientific, A39255), 1× lithium dodecyl sulfate (LDS) sample buffer (Invitrogen, NP0007), and 20 mM iodoacetamide (Thermo Fisher Scientific, A39271), and heated at 95°C for 15 min. The alum-bound protein in the pellet was desorbed by resuspending the pellet in an elution buffer (0.4 M sodium phosphate, 50 mM dithiothreitol, 1× LDS sample buffer, and 20 mM iodoacetamide) and heating the samples at 95°C for 15 min. The desorbed samples were then centrifuged at 4000*g* for 10 min at 4°C to obtain desorbed protein in the supernatant for SDS-PAGE. RBD-SpyTag and HBsAg-SpyCatcher controls were prepared for SDS-PAGE similar to unbound protein sample. Approximately 0.1 μg of each sample was loaded on 4 to 12% bis-tris gel (Invitrogen, NP3022) run for 50 min at 150 V in 1× MES-SDS running buffer (Invitrogen, NP0002). Novex Sharp Pre-stained protein standard (Invitrogen, LC5800) was used as marker to estimate molecular weight of the proteins. The gel was stained using a Pierce Silver Stain kit (Thermo Fisher Scientific, 24612) as per manufacturer’s protocol and imaged using ProteinSimple FluorChem E imaging system.

### Competitive ELISA by ACE2-Fc binding

Stock adjuvanted RBD-VLP samples were first incubated with a blocking buffer, and then twofold serial dilutions were made followed by incubation with ACE2-Fc receptor (0.02 mcg/ml) overnight on a plate rotator at room temperature. Corresponding blank samples were prepared using blocking buffer alone, while the saturated samples contained ACE2-Fc receptor. Samples were centrifuged the next day, and the supernatant containing unbound ACE2-Fc was transferred to a 96-well plate coated with RBD (1 mcg/ml). The plate was incubated for 2 hours at 25°C, washed, and the amount of bound ACE2-Fc on the plate was detected with a horseradish peroxidase (HRP)–conjugated secondary antibody using a tetramethylbenzidine substrate. The percent relative ACE2-Fc binding of samples was determined from the optical density at 450 nm values using the parameters obtained from a four-point logistic fit of the standard run on each plate.

### Production of unformulated drug substance

HBsAg SpyCatcher nanoparticles were produced in a GMP process at the Serum Institute of India. RBD-SpyTag protein was expressed in shake flask culture of *P. pastoris* and purified as described previously (*26*, *30*). HBsAg-SpyCatcher and RBD-SpyTag were conjugated by incubation overnight at 4°C in 20 mM sodium phosphate and 150 mM NaCl (pH 8) buffer, with a 1:1.5 HBsAg:RBD molar ratio. Excess RBD was removed with a 100-kDa molecular weight cutoff Amicon Ultra-4 centrifugal filter (Millipore).

### Biolayer interferometry

Biolayer interferometry was performed using the Octet Red96 with Protein A biosensors (Sartorius ForteBio, Fremont, CA), which were hydrated for 15 min in kinetics buffer before each run. Kinetics buffer comprising 1× phosphate-buffered saline (PBS) (pH 7.2), 0.5% bovine serum albumin, and 0.05% Tween 20 was used for all dilutions, baseline, and disassociation steps. CR3022 and ACE2-Fc were used in the assay at concentrations of 2 and 10 μg/ml, respectively. Samples were loaded in a 96-well black microplate (Greiner Bio-One, Monroe, NC) at starting concentrations of 15 and 10 μg/ml, respectively. Seven 1:1 serial dilutions and a reference well of kinetics buffer were analyzed for each sample. Association and dissociation were measured at 1000 rpm for 300 and 600 s, respectively. Binding affinity was calculated using the Octet Data Analysis software v10.0 (Pall ForteBio), using reference subtraction, baseline alignment, interstep correction, Savitzky-Golay filtering, and a global 1:1 binding model.

### Negative-stain EM

Solution with conjugated RBD-VLP nanoparticles (7 μl) was incubated on a 200-mesh copper grid coated with a continuous carbon film for 60 s. Excess liquid was removed, and the film was incubated in 10 μl of 2% uranyl acetate. The grid was dried at room temperature and mounted on a JEOL single-tilt holder in the transmission EM column. The specimen was cooled by liquid nitrogen and imaged on a JEOL 2100 Field Emission Gun microscope with a minimum dose to avoid sample damage. The microscope was operated at 200 kV with magnification of ×10,000 to ×60,000. Images were recorded on a Gatan 2Kx2K UltraScan charge-coupled device camera.

### Immunization of nonhuman primates

Eighteen cynomolgus macaques were randomly allocated to three groups of six animals each. Animals were housed at Bioqual Inc. (Rockville, MD). On day 0, groups of animals were immunized with: (i) RBD-HBsAg VLP containing 5 μg total protein adjuvanted with 500 μg alum, (ii) RBD-HBsAg VLP containing 5 μg protein adjuvanted with 500 μg of alum + 1500 μg CpG 1018, or (iii) sham. Animals were administered an identical boost immunization of the regimens as indicated above at week 3. At week 6, all animals were challenged with 1 × 10^5^ median tissue culture infectious dose of SARS-CoV-2 via the intranasal and intratracheal routes (2 ml total volume per animal; 1 ml intranasal + 1 ml intratracheal). The challenge stock was derived from a single passage of a SARS-CoV-2 2019 USA-WA1/2020 isolate (BEI Resources, NR-53780). All animal studies were conducted in compliance with all relevant local, state, and federal regulations. All experiments were reviewed and approved by the BIOQUAL Institution Animal Care and Use Committee.

### ELISA assays

Binding antibodies were quantified by ELISA essentially as previously described (*45*). Briefly, 96-well plates were coated with SARS-CoV-2 S protein (1 μg/ml; Sino Biological) in 1× Dulbecco’s phosphate buffered saline (DPBS) and incubated overnight at 4°C. Plates were then washed once with wash buffer (0.05% Tween 20 in 1 × DPBS) and blocked for 2 to 3 hours with 350 μl of casein block per well at room temperature. After blocking, serial dilutions of serum diluted in casein block were added to wells, and plates were incubated for 1 hour at room temperature. Plates were then washed three times, a dilution (1 μg/ml) of anti-macaque immunoglobulin G HRP (Nonhuman Primate Reagent Resource) was added to wells, and plates were incubated at room temperature in the dark for 1 hour. Plates were again washed three times, and then 100 μl of SeraCare KPL trimethylboron (TMB) SureBlue Start solution was added to each well. Plate development was halted with the addition of 100 μl of SeraCare KPL TMB Stop solution per well. A VersaMax microplate reader was used to record the absorbance at 450 nm. For each sample, end point titer was calculated in Graphpad Prism software using a four-parameter logistic curve fit to calculate the reciprocal serum dilution that yields an absorbance value of 0.2 at 450 nm. Log_10_ end point titers are reported.

### Pseudovirus neutralization assays

SARS-CoV-2 pseudoviruses were generated essentially as described previously (*45*, *46*). Briefly, human embryonic kidney (HEK) 293T cells were cotransfected with: (i) the packaging plasmid psPAX2 (AIDS Resource and Reagent Program), (ii) luciferase reporter plasmid pLenti-CMV Puro-Luc (Addgene), and (iii) an S protein–expressing plasmid, pcDNA3.1-SARS-CoV-2 SΔCT using Lipofectamine 2000 (Thermo Fisher Scientific). This approach was used to generate pseudoviruses specific to SARS-CoV-2 variant strains, including WA1/2020 strain (Wuhan/WIV04/2019, Global Initiative on Sharing Avian Influenza Data (GISAID) accession ID: EPI_ISL_402124), D614G mutation, B.1.1.7 variant (GISAID accession ID: EPI_ISL_601443), and B.1.351 variant (GISAID accession ID: EPI_ISL_712096). To purify pseudoviruses, supernatants were collected 48 hours after transfection, centrifuged, and then passed through a 0.45-μm filter. To determine the neutralization activity of the serum samples from nonhuman primates, HEK293T-hACE2 cells were seeded in 96-well tissue culture plates at a density of 1.75 × 10^4^ cells per well and incubated overnight. Serial dilutions of heat-inactivated serum samples were prepared and mixed with 50 μl of indicated pseudovirus. This mixture was incubated at 37°C for 1 hour before adding to seeded HEK293T-hACE2 cells. Forty-eight hours after infection, cells were lysed in Steady-Glo Luciferase Assay Reagent (Promega) according to the manufacturer’s instructions. SARS-CoV-2 neutralization titers were defined as the sample dilution at which a 50% reduction in relative light unit was observed relative to the average of the virus control wells.

### ELISPOT assays

Enzyme-linked immune absorbent spot (ELISPOT) assays were performed essentially as described previously (*45*). Plates were coated with mouse anti-human IFN-γ antibody (BD Pharmigen) at 5 μg per well and incubated at 4°C overnight. After incubation, plates were washed with wash buffer (1× DPBS with 0.25% Tween 20) and then blocked with R10 media (RPMI 1640 with fetal bovine serum and penicillin-streptomycin) for 1 to 4 hours at 37°C. SARS-CoV-2 S1 peptides were prepared and plated at 1 μg per well. A total of 200,000 cells per well were added to the plate and incubated for 18 to 24 hours at 37°C. Plates were then washed with wash buffer (MilliQ water with DPBS and Tween 20) and incubated for 2 hours at room temperature with polyclonal rabbit anti-human IFN-γ biotin from U-Cytech at 1 μg/ml. The plates were washed again and then incubated for 2 hours at room temperature with streptavidin-alkaline phosphatase from Southern Biotech at 2 μg/ml. A final wash step was followed by the addition of nitro-blue tetrazolium chloride–5-bromo-4-chloro 3 ′indolyphosphate p-toludine salt (NBT/BCIP chromogen) substrate solution for 7 min. The chromogen was discarded, and the plates were washed with water and dried for 24 hours protected from light. Plates were scanned and counted on a Cellular Technologies Limited Immunospot Analyzer.

### Viral load assays

SARS-CoV-2 E gene sgRNA was assessed by reverse transcription polymerase chain reaction (RT-PCR) using primers and probes essentially as previously described (*45*). To generate a standard, a fragment of the subgenomic E gene was synthesized and cloned into a pcDNA3.1+ expression plasmid using restriction site cloning (Integrated DNA Techonologies). The insert was in vitro–transcribed to RNA using the AmpliCap-Max T7 High Yield Message Maker Kit (CellScript). Log-fold dilutions were prepared for RT-PCR assays, ranging from 1 × 10^10^ to 1 × 10^−1^ copies. To quantify viral loads in respiratory tract tissues, samples of BAL fluid and nasal swabs were analyzed. RNA extraction was performed on a QIAcube HT using the IndiSpin QIAcube HT Pathogen Kit according to manufacturer’s specifications (Qiagen). Standards described above and extracted RNA from nonhuman primate samples were reverse-transcribed using SuperScript VILO Master Mix (Invitrogen), using the cycling conditions specified by the manufacturer, 25°C for 10 min, 42°C for 1 hour, and then 85°C for 5 min. A Taqman custom gene expression assay (Thermo Fisher Scientific) was designed using the sequences targeting the E gene sgRNA. The sequences for the custom assay were as follows: forward primer, sgLeadCoV2.Fwd: CGATCTCTTGTAGATCTGTTCTC, E_Sarbeco_R: ATATTGCAGCAGTACGCACACA, E_Sarbeco_P1 (probe): VIC-ACACTAGCCATCCTTACTGCGCTTCG-MGB. Reactions were carried out in duplicate for samples and standards on the QuantStudio 6 and 7 Flex Real-Time PCR Systems (Applied Biosystems) with the following thermal cycling conditions: initial denaturation at 95°C for 20 s, followed by 45 cycles of 95°C for 1 s, and 60°C for 20 s. Standard curves were used to calculate sgRNA copies per milliliter of BAL fluid or per swab; the quantitative assay sensitivity was 50 copies/ml or per swab.

### Histopathology

At time of fixation, lungs were suffused with 10% formalin to expand the alveoli. All tissues were fixed in 10% formalin and blocks sectioned at 5 μm. Slides were baked for 30 to 60 min at 65°, deparaffinized in xylene, rehydrated through a series of graded ethanol to distilled water, and then stained with hematoxylin and eosin. Blinded histopathological evaluation was performed by a board-certified veterinary pathologist (A.J.M.). Two lung lobes (one section from the right and left caudal lung lobes) were assessed and scored (1 to 4) for each of the following lesions: (i) interstitial inflammation and septal thickening, (ii) eosinophilic interstitial infiltrate, (iii) neutrophilic interstitial infiltrate, (iv) hyaline membranes, (v) interstitial fibrosis, (vi) alveolar infiltrate and macrophage, (vii) alveolar/bronchoalveolar infiltrate and neutrophils, (viii) syncytial cells, (ix) type II pneumocyte hyperplasia, (x) broncholar infiltrate and macrophage, (xi) broncholar infiltrate and neutrophils, (xii) bronchus-associated lymphoid tissue (BALT) hyperplasia, (xiii) bronchiolar/peribronchiolar inflammation, (xiv) perivascular and mononuclear infiltrates, and (xv) vessels and endothelialitis. Each feature assessed was assigned a score of 0 = no significant findings; 1 = minimal; 2 = mild; 3 = moderate; and 4 = marked/severe.

## Notes

View/request a protocol for this paper from *Bio-protocol*.
