# Supplementary material for: SARS-CoV-2 receptor binding domain displayed on HBsAg virus–like particles elicits protective immunity in macaques
Source: Sci Adv. 2022 Mar 16;8(11):eabl6015. doi: 10.1126/sciadv.abl6015 (PMC8926328; doi:10.1126/sciadv.abl6015)
Supplement: Supplementary file 1 — Figs. S1 and S2 [file sciadv.abl6015_sm.pdf]

Science Advances
AAAS

Supplementary Materials for

SARS-CoV-2 receptor binding domain displayed on HBsAg virus–like particles elicits protective immunity in macaques

Neil C. Dalvie, Lisa H. Tostanoski, Sergio A. Rodriguez-Aponte, Kawaljit Kaur, Sakshi Bajoria, Ozan S. Kumru, Amanda J. Martinot, Abishek Chandrashekar, Katherine McMahan, Noe B. Mercado, Jingyou Yu, Aiquan Chang, Victoria M. Giffin, Felix Nampanya, Shivani Patel, Lesley Bowman, Christopher A. Naranjo, Dongsoo Yun, Zach Flinchbaugh, Laurent Pessaint, Renita Brown, Jason Velasco, Elyse Teow, Anthony Cook, Hanne Andersen, Mark G. Lewis, Danielle L. Camp, Judith Maxwell Silverman, Gaurav S. Nagar, Harish D. Rao, Rakesh R. Lothe, Rahul Chandrasekharan, Meghraj P. Rajurkar, Umesh S. Shaligram, Harry Kleanthous, Sangeeta B. Joshi, David B. Volkin, Sumi Biswas, J. Christopher Love*, Dan H. Barouch*

*Corresponding author. Email: dbarouch@bidmc.harvard.edu (D.H.B.); clove@mit.edu (J.C.L.)

Published 16 March 2022, *Sci. Adv.* **8**, eabl6015 (2022)
DOI: 10.1126/sciadv.abl6015

This PDF file includes:

Figs. S1 and S2

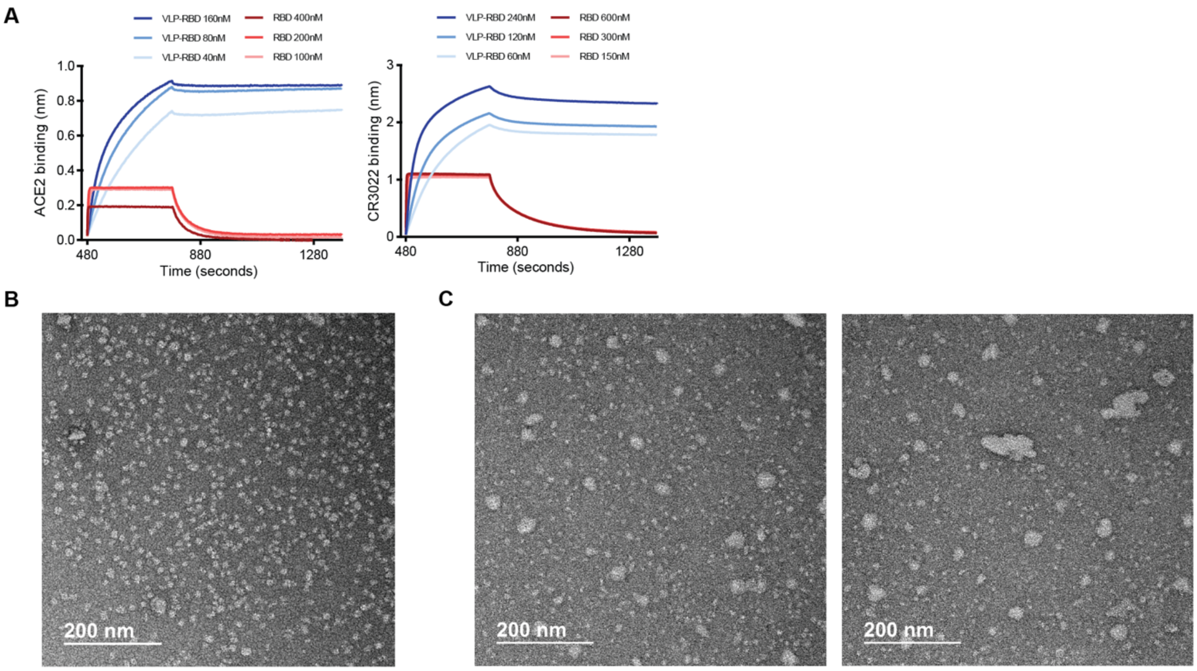

Fig. S1. Analysis of representative samples of RBD-VLP antigen

A) Biolayer interferometry of binding to human ACE2-Fc protein, and CR3022 neutralizing antibody. RBD-VLP conjugated protein is shown in blue. RBD-spytag monomer is shown in red. **B-C**) Negative stain electron microscopy of unconjugated HBsAg-VLP (**B**) and conjugated RBD-VLP (**C**).

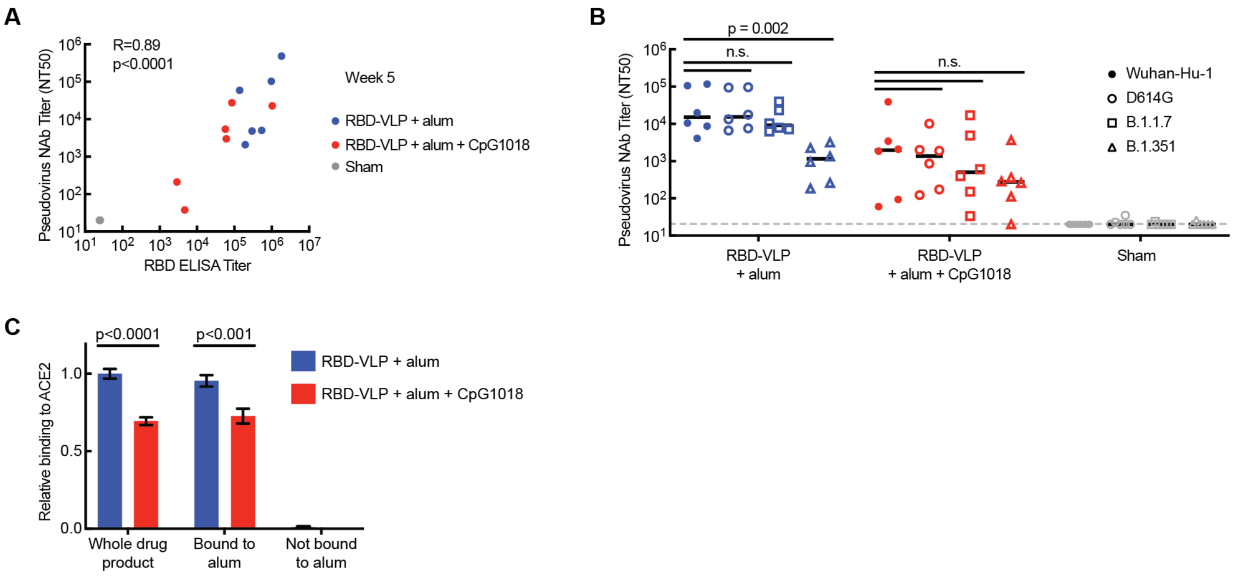

Fig. S2. Further analysis of the humoral immune response

A) Correlation of RBD-specific antibody titer and pseudovirus neutralizing antibody titer from week 5 animal sera. R was calculated by Spearman correlation. **B**) Titers of neutralizing antibodies to SARS-CoV-2 variant pseudoviruses in animal sera. Statistical significance was determined by a Kolmogorov-Smirnov test. N.S. = not significant ($p > 0.1$). Black bars represent median values. Dotted gray line represents limit of detection. **C**) Relative binding of adjuvanted RBD-VLP to ACE2-Fc by competitive ELISA. Alum-bound protein was separated to bound and unbound only fractions by centrifugation. Error represents standard deviation after two independent measurements.
